# Clinicomics-guided distant metastasis prediction in breast cancer via artificial intelligence

**DOI:** 10.1186/s12885-023-10704-w

**Published:** 2023-03-14

**Authors:** Chao Zhang, Lisha Qi, Jun Cai, Haixiao Wu, Yao Xu, Yile Lin, Zhijun Li, Vladimir P. Chekhonin, Karl Peltzer, Manqing Cao, Zhuming Yin, Xin Wang, Wenjuan Ma

**Affiliations:** 1 Tianjin Medical University Cancer Institute and Hospital, National Clinical Research Center for Cancer, Key Laboratory of Cancer Prevention and Therapy, Tianjin’s Clinical Research Center for Cancer, Tianjin, China; 2 The Sino-Russian Joint Research Center for Bone Metastasis in Malignant Tumor, Tianjin, China; 3 Tianjin Medicine and Health Research Center, Tianjin Institute of Medical & Pharmaceutical Sciences, Tianjin, China; 4 Department of Basic and Applied Neurobiology, Federal Medical Research Center for Psychiatry and Narcology, Moscow, Russian Federation; 5 Department of Psychology, University of the Free State, Turfloop, South Africa; 6 Department of Epidemiology and Biostatistics, West China School of Public Health, Sichuan University, Chengdu, Sichuan Province China

**Keywords:** Artificial Intelligence, Prediction, Image, Metastasis, Breast Cancer

## Abstract

**Background:**

Breast cancer has become the most common malignant tumour worldwide. Distant metastasis is one of the leading causes of breast cancer-related death. To verify the performance of clinicomics-guided distant metastasis risk prediction for breast cancer via artificial intelligence and to investigate the accuracy of the created prediction models for metachronous distant metastasis, bone metastasis and visceral metastasis.

**Methods:**

We retrospectively enrolled 6703 breast cancer patients from 2011 to 2016 in our hospital. The figures of magnetic resonance imaging scanning and ultrasound were collected, and the figures features of distant metastasis in breast cancer were detected. Clinicomics-guided nomogram was proven to be with significant better ability on distant metastasis prediction than the nomogram constructed by only clinical or radiographic data.

**Results:**

Three clinicomics-guided prediction nomograms on distant metastasis, bone metastasis and visceral metastasis were created and validated. These models can potentially guide metachronous distant metastasis screening and lead to the implementation of individualized prophylactic therapy for breast cancer patients.

**Conclusion:**

Our study is the first study to make cliniomics a reality. Such cliniomics strategy possesses the development potential in artificial intelligence medicine.

**Supplementary Information:**

The online version contains supplementary material available at 10.1186/s12885-023-10704-w.

## Introduction

Breast cancer (BC) has become the most common malignant tumour worldwide, its incidence was reported to increase by approximately 0.5% yearly [1]. The 5-year survival of early-stage BC patients is approximately 95%; once distant metastasis (DM) occurs, prognosis significantly deteriorates [2, 3]. Accurate identification of BC patients at high risk for DM risk, prophylactic treatment and close follow-up could improve the prognosis of BC patients. An earlier window for treatment can potentially be created with the identification of BC patients at high metastatic risk.

Prior studies predicted DM in BC patients with established mathematical models [4, 5]. Currently, most models are created based only on clinical or radiographic data. Artificial intelligence (AI) guided models are known to have the potential for wide application [6–8]. The clinicomics approach involves multiple disease features that are routinely evaluated [9, 10]. Such features include complete history, epidemiological distribution, physical examination, laboratory testing, imaging evaluation, and histological examination. We hypothesized that the incorporation of multidimensional data into the prediction model could result in a deeper understanding of the disease and a higher prognostic prediction accuracy. No studies have been conducted to validate this hypothesis since it was first described in 2005. Data dimension reduction before the application of AI was the main dilemma for the validation and development of clinicomics methods. Currently, radiomics and deep learning can comprehensively analyse features from imaging and even from videos, making clinicomics a potential reality.

In the present study, we aimed to verify the performance of clinicomics-guided prognostic prediction for breast cancer via artificial intelligence and to investigate the accuracy of the created prediction models for metachronous DM, bone metastasis and visceral metastasis. These models can potentially guide metachronous DM screening and lead to the implementation of individualized prophylactic therapy for BC patients with a high risk for DM.

## Materials and methods

### Study design and participants

**Fig. 1:**
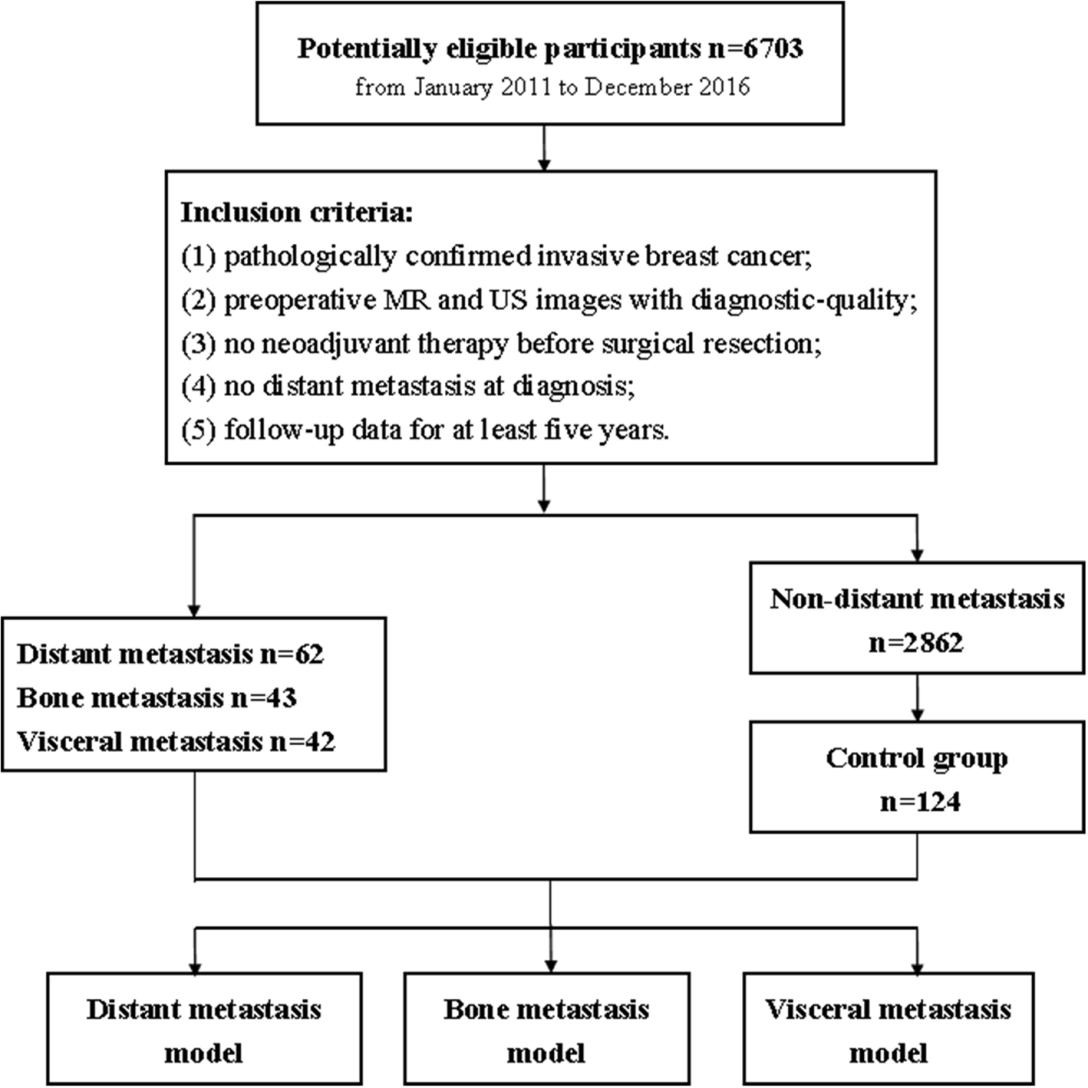
The flowchart of the proposed distant metastasis prediction system

This case–control study protocol was approved by the Ethics Committee of Tianjin Medical University Cancer Institute & Hospital, Tianjin, China (EK2018125). A total of 6,703 consecutive BC patients from the hospital between January 2011 and December 2016 were included. The detailed inclusion and exclusion criteria were as follows: (1) a histopathological diagnosis of invasive BC through surgically resected specimens and/or needle biopsy; (2) availability of diagnostic-quality preoperative magnetic resonance imaging (MRI) scanning and ultrasound (US) images; (3) MRI scanning and US exam conducted before neoadjuvant therapy or surgical resection; (4) no DM present at diagnosis; and (5) follow-up data available for at least five years. Sixty-two patients with DM and 124 randomly selected patients without DM were included in the present study. The flow chart of the present study is shown in Fig. 1. The patients’ demographic and clinicopathological characteristics were collected from their medical records (Table 1). To validate the performance of the prediction model, the included patients were randomly divided into two sets: the training set (70%, *N* = 131) and the validation set (30%, *N* = 55).

**Table 1:** Clinicopathologic characteristics between breast cancer patients with /without distant metastasis

Character	Training set				Validation set	
	Distant metastasis		HR (95% CI)	*P*-value	Distant metastasis	
	no	yes			no	yes
Age (mean ± SD, years)	47.4 (9.23)	49.7 (9.96)	1.020 (0.991–1.054)	0.166	48.3 (10.8)	42.5 (13.6)
Family history of BC						
Yes	3 (3.41)	2 (4.65)	1.255 (0.304–5.192)	0.754	3 (8.33%)	0 (0.00%)
No	85 (96.59)	41 (95.35)			33 (91.67%)	19 (100.00%)
Breast-feeding histories						
Yes	77 (87.50)	35 (81.40)	0.731 (0.339–1.575)	0.423	31 (86.11%)	15 (78.95%)
No	11 (12.50)	8 (18.60)			5 (13.89%)	4 (21.05%)
Abortion						
Yes	55 (62.50)	21 (48.84)	0.639 (0.3513–1.162)	0.142	21 (58.33%)	10 (52.63%)
No	33 (37.50)	22 (51.16)			15 (41.67%)	9 (47.37%)
Reproductive history						
Yes	85 (96.59)	38 (88.37)	0.455 (0.179–1.157)	0.098	33 (91.67%)	16 (84.21%)
No	3 (3.41)	5 (11.63)			3 (8.33%)	3 (15.79%)
Menstrual status						
Menstruate	57 (64.77)	23 (53.49)	1.503 (0.825–2.738)	0.183	21 (58.33%)	14 (73.68%)
Menopause	31 (35.23)	20 (46.51)			15 (41.67%)	5 (26.32%)
Age of menarche	14.6 (1.71)	14.3 (2.03)	0.9402 (0.7914–1.117)		14.4 (1.78)	14.8 (1.69)
Lymph node metastasis				0.484		
Have	18 (20.45)	26 (60.47)	4.197 (2.27–7.759)	< 0.001	12 (33.33%)	11 (57.89%)
None	70 (79.55)	17 (39.53)			24 (66.67%)	8 (42.11%)
molecular subtyping						
1	9 (10.23)	5 (11.63)	1.109 (0.8039–1.53)	0.529	4 (11.11%)	2 (10.53%)
2	59 (67.05)	23 (53.49)			26 (72.22%)	10 (52.63%)
3	5 (5.68)	6 (13.95)			1 (2.78%)	3 (15.79%)
4	15 (17.05)	9 (20.93)			5 (13.89%)	4 (21.05%)
ER						
Positive	68 (77.27)	27 (62.79)	0.634 (0.3412–1.178)	0.149	30 (83.33%)	10 (52.63%)
Negative	20 (22.73)	16 (37.21)			6 (16.67%)	9 (47.37%)
PR						
Positive	65 (73.86)	25 (58.14)	0.6001 (0.3271–1.101)	0.099	30 (83.33%)	10 (52.63%)
Negative	23 (26.14)	18 (41.86)			6 (16.67%)	9 (47.37%)
HER2 status						
Positive	27	17	1.09 (0.735–1.616)	0.669	23	10
Negative	61	26			13	9
Ki-67						
Positive	78 (88.64)	38 (88.37)	1.003 (0.991–1.016)	0.600	31 (86.11%)	16 (84.21%)
Negative	10 (11.36)	5 (11.63)			5 (13.89%)	3 (15.79%)
TPSA						
Positive	10 (11.36)	6 (13.95)	1.268 (0.5349–3.006)	0.59	3 (8.33%)	3 (15.79%)
Negative	78 (88.64)	37 (86.05)			33 (91.67%)	16 (84.21%)
CA153						
Positive	1 (1.14)	10 (23.26)	7.407 (3.573–15.36)	< 0.001	0 (0.00%)	4 (21.05%)
Negative	87 (98.86)	33 (76.74)			36 (100.00%)	15 (78.95%)
CEA						
Positive	0 (0.00)	9 (20.93)	9.77 (4.489–21.26)	< 0.001	1 (2.78%)	3 (15.79%)
Negative	88 (100.00)	34 (79.07)			35 (97.22%)	16 (84.21%)
CA125						
Positive	4 (4.55)	8 (18.60)	3.195 (1.479–6.9)	0.003	4 (11.11%)	2 (10.53%)
Negative	84 (95.45)	35 (81.40)			32 (88.89%)	17 (89.47%)
Operation						
No surgery	0 (0.00)	7 (16.28)	0.8021 (0.4741–1.357)	0.411	0 (0.00%)	0 (0.00%)
Conserving	39 (44.32)	8 (18.60)			16 (44.44%)	7 (36.84%)
Radical	49 (55.68)	28 (65.12)			20 (55.56%)	12 (63.16%)
Endocrinotherapy						
Yes	11 (12.50)	0 (0.00)	1.281e-08 (0-Inf)	0.996	1 (2.78%)	0 (0.00%)
No	77 (87.50)	43 (100.00)			35 (97.22%)	19 (100.00%)
Radiotherapy						
Yes	13 (14.77)	10 (23.25)	1.476 (0.7272–2.997)	0.281	4 (11.11%)	4 (21.05%)
No	75 (85.23)	33 (76.74)			32 (88.89%)	15 (78.95%)
Chemotherapy						
Yes	79 (89.77)	42 (97.67)	4.124 (0.5675–29.97)	0.161	34 (94.44%)	19 (100.00%)
No	9 (10.23)	1 (2.33)			2 (5.56%)	0 (0.00%)
RadScore (mean ± SD)	-2.84 (0.49)	-1.67 (0.46)	l	< 0.001	-2.48 (0.72)	-2.42 (0.75)

*Abbreviations: ER* Expression of the oestrogen receptor, *PR* Progesterone receptor, *HER2* Human epidermal growth factor receptor 2, *TPSA* Total prostate-specific antigen, *CA125* Carbohydrate antigen 125, *CEA* Carcinoembryonic antigen, *CA153* Carbohydrate antigen 125

### MRI and US technique

All patients underwent MRI and US examinations within 2 weeks before breast surgery. Magnetic resonance images were acquired using scanners manufactured by two companies, a 1.5-T system (Signa Infinity Excite II, GE Healthcare) before 2013, and a 3.0-T MRI system (Discovery MR750, GE Medical Systems) after 2013. The detailed MRI parameters are shown in the Supplementary Materials and Methods. All ultrasound images were acquired using a GE LOGIQ7 or GE LOGIQ E9 ultrasound machine with a 6 ~ 15.0 MHz probe.

### Imaging feature detection and radiomics signature construction

MRI and US images were retrieved from picture archiving and communication systems (PACS) for image segmentation and analysis. The lesions were segmented by a radiologist with more than 8 years of experience using ImageJ (https://imagej.nih.gov/ij/). Another experienced radiologist (with 30 years of experience) was consulted when the lesion boundaries in US were not determined clearly.

A total of 2569 radiomics features (855 features from magnetic resonance T2 weighted images (T2WI), 859 features from dynamic-contrast enhanced MRI (DCE-MRI) and 855 from US images) were extracted for each patient. Detailed information about the feature extraction algorithms is provided in Supplementary Table S1. The feature extraction method was performed using in-house software written with MATLAB R2018b (MathWorks, Inc., Natick, Massachusetts).

In order to test the prediction ability of different image types, we built models based on each type and a model based on the integrated features from all types. We followed a three-step procedure to determine reliable radiomic features. First, the Wilson test was used to identify features that were highly correlated to the biomarkers with a significant value (*P* < 0.05). Pearson correlation matrices were used to evaluate the correlation between the features, and the correlation coefficient greater than 0.8 was considered redundant. One of two features with a lower *P*-value was excluded. Subsequently, the optimal prognostic combination of features was selected by using the minor absolute contraction and Selection operator (LASSO) regression method. By calculating the radial score (radscore), the linear combination of each patient's selected features weighted by their respective coefficients was calculated to establish the prediction model [11]. A fixed 70%/30% training/rest set split was used, and tenfold cross-validation was performed to assess the true diagnostic potential of the model.

### The clinicopathology and the clinicomics-based nomograms

Univariate analysis was used to evaluate the clinicopathological factors in the training set. Variables with *P* < 0.05 of univariate analysis was included in the Cox proportional hazards regression model, and the clinicopathological nomogram was established to predict DM risk in BC. We evaluated clinicomics-based nomogram to determine whether the model has the best performance in predicting DM risk in BC.

### Statistical analysis

Continuous variables are expressed as the mean ± standard deviation (normally distributed) or median with interquartile range (abnormally distributed), while categorical variables are expressed as numbers and percentages. The predictive accuracy of nomogram was evaluated by the area under the receiver operating characteristic (ROC) curve and Harrell’s concordance index (C-index), while the calibration ability was evaluated by calibration curves. The difference in the area under the curve (AUC) between the training and validation datasets was tested by the *P*-value of Delong’s test. The integrated discrimination improvement (IDI) values were assessed to quantify the incremental prognostic improvement in the radiomic signature. The statistical analyses were conducted using R software (version 6.1, R Foundation for Statistical Computing, Vienna, Austria). A two-tailed difference with *P* < 0.05 was considered significant. The packages used in the current study included glmnet, time ROC, rms, survival, Hmisc and rmda.

## Results

### Characteristics of distant metastasis

The clinicopathological characteristics of the training (*n* = 131) and test (*n* = 55) sets are summarized in Table 1. The median incubation time of BC patients with DM was 14 months (range, 1–58 months). visceral (*N* = 42) was the most frequent metastasis site, followed by bone (*N* = 33) and brain (*N* = 10) sites.

### Patient clinical characteristics and development of the clinical factor DM model

Among clinicopathologic characteristics, lymph node metastasis (*P* < 0.001), higher levels of CA153 (*P* < 0.001), carcinoma embryonic antigen (CEA) (*P* < 0.001) and CA125 (*P* = 0.003) were significantly associated with DM risk among BC patients in the training set (Table 1), and these factors were used to establish the clinicopathological model (Supplementary Figure S1). Subgroup analysis of age (younger than 50 years / older than 50 years) to predict DM was showed in Supplementary Table S2. Reproductive history (*P* = 0.036), lymph node metastasis (*P* < 0.001), and higher levels of CA153 (*P* < 0.001), CEA (*P* < 0.001) and CA125 (*P* = 0.015) were associated with bone metastasis risk (Table 2). Lymph node metastasis (*P* = 0.001), oestrogen receptor (ER)-positive status (*P* < 0.001), progesterone receptor (PR)-positive status (*P* < 0.001), higher levels of CA153 (*P* < 0.001), CEA (*P* = 0.004) and endocrinotherapy (*P* = 0.041) were associated with viscera metastasis risk (Table 3). ER positivity (*P* = 0.005) and higher levels of CA153 (*P* < 0.001), CEA (*P* < 0.001) and CA125 (*P* = 0.009) were associated with brain metastasis risk (Table 4).

**Table 2:** Clinicopathologic characteristics between breast cancer patients with/without bone metastasis

Character	Training set				Validation set	
	Distant metastasis		HR (95% CI)	*P*-value	Distant metastasis	
	no	yes			no	yes
Age (mean ± SD, years)	46.8 (9.75)	47.1 (12.3)	1 (0.964–1.05)	0.847	49.6 (9.27)	45.6 (7.82)
Family history of BC						
Yes	5 (5.75)	0 (0.00)	< 0.001 (0-Inf)	0.997	1 (2.70%)	1 (10.00%)
No	82 (94.25)	23 (100.00)			36 (97.30%)	9 (90.00%)
Breast-feeding histories						
Yes	76 (87.36)	18 (78,326)	0.579 (0.215–1.56)	0.279	32 (86.49%)	8 (80.00%)
No	11 (12.64)	5 (21.74)			5 (13.51%)	2 (20.00%)
Abortion						
Yes	52 (59.77)	11 (47.83)	0.622 (0.274–1.41)	0.255	24 (64.86%)	6 (60.00%)
No	35 (40.23)	12 (52.17)			13 (35.14%)	4 (40.00%)
Reproductive history						
Yes	83 (95.40)	19 (82.61)	0.314 (0.107–0.926)	0.036	35 (94.59%)	9 (90.00%)
No	4 (4.60)	4 (17.39)			2 (5.41%)	1 (10.00%)
Menstrual status						
Menstruate	57 (65.52)	15 (65.22)	1.05 (0.446–2.48)	0.908	21 (56.76%)	7 (70.00%)
Menopause	30 (34.48)	8 (34.78)			16 (43.24%)	3 (30.00%)
Age of menarche	14.1 (1.73)	14.6 (1.73)	1.04 (0.82–1.32)	0.741	14.7 (1.73)	14.2 (2.66)
Lymph node metastasis						
Have	19 (21.84)	14 (60.87)	4.76 (2.06–11)	< 0.001	11 (29.73%)	7 (70.00%)
None	68 (78.16)	9 (39.13)			26 (70.27%)	3 (30.00%)
molecular subtyping						
1	9 (10.34)	4 (17.39)	0.738 (0.419–1.3)	0.293	4 (10.81%)	0 (0.00%)
2	61 (70.11)	16 (69.57)			24 (64.86%)	7 (70.00%)
3	4 (4.60)	1 (4.35)			2 (5.41%)	1 (10.00%)
4	13 (14.94)	2 (8.70)			7 (18.92%)	2 (20.00%)
ER						
Positive	70 (80.46)	19 (82.61)	1.2 (0.407–3.52)	0.743	28 (75.68%)	7 (70.00%)
Negative	17 (19.54)	4 (17.39)			9 (24.32%)	3 (30.00%)
PR						
Positive	68 (78.16)	20 (86.96)	1.8 (0.535–6.06)	0.343	27 (72.97%)	4 (40.00%)
Negative	19 (21.84)	3 (13.04)			10 (27.03%)	6 (60.00%)
HER2 status						
Positive	57	15	0.931 (0.528–1.64)	0.805	27	6
Negative	30	8			10	4
Ki-67						
Positive	76 (87.36)	20 (86.96)	1 (0.983–1.02)	0.985	33 (89.19%)	9 (90.00%)
Negative	11 (12.64)	3 (13.04)			4 (10.81%)	1 (10.00%)
TPSA						
Positive	9 (10.34)	5 (21.74)	2.13 (0.788–5.73)	0.136	4 (10.81%)	1 (10.00%)
Negative	78 (89.66)	18 (78.26)			33 (89.19%)	9 (90.00%)
CA153						
Positive	1 (1.15)	7 (30.43)	13.4 (5.27–33.8)	< 0.001	0 (0.00%)	1 (10.00%)
Negative	86 (98.85)	16 (69.57)			37 (100.00%)	9 (90.00%)
CEA						
Positive	0 (0.00)	4 (17.)	39.9 (10.7–149)	< 0.001	1 (2.70%)	2 (20.00%)
Negative	87 (100.00)	19 (82.61)			36 (97.30%)	8 (80.00%)
CA125						
Positive	3 (3.45)	4 (17.39)	4.37 (1.48–12.9)	0.008	5 (13.51%)	2 (20.00%)
Negative	84 (96.55)	19 (82.61)			32 (86.49%)	8 (80.00%)
Operation						
No surgery	0 (0.00)	3 (13.04)	0.597 (0.282–1.26)	0.178	0 (0.00%)	3 (30.00%)
Conserving	39 (44.83)	8 (34.78)			16 (43.24%)	2 (20.00%)
Radical	48 (55.17)	12 (52.17)			21 (56.76%)	5 (50.00%)
Endocrinotherapy						
Yes	10 (11.49)	0 (0.00)	1.28e-08 (0-Inf)	0.997	2 (5.41%)	0 (0.00%)
No	77 (88.51)	23 (100.00)			35 (94.59%)	10 (100.00%
Radiotherapy						
Yes	12 (13.79)	6 (26.09)	0.105	0.159	5 (13.51%)	5 (50.00%)
No	75 (86.21)	17 (73.91)			32 (86.49%)	5 (50.00%)
Chemotherapy						
Yes	80 (91.95)	23 (100.00)	1.91 (0.754–4.86)	0.172	33 (89.19%)	10 (100.00%)
No	7 (8.05)	0 (0.00)			4 (10.81%)	0 (0.00%)
RadScore (mean ± SD)	-2.77 (0.56)	-1.72 (0.43)	15.9 (6.43–39.5)	< 0.001	-2.60 (0.67)	-2.40 (0.71)

*Abbreviations: ER* Expression of the oestrogen receptor, *PR* Progesterone receptor, *HER2* Human epidermal growth factor receptor 2, *TPSA* Total prostate-specific antigen, *CA125* Carbohydrate antigen 125, *CEA* Carcinoembryonic antigen, *CA153* Carbohydrate antigen 125

**Table 3:** Clinicopathologic characteristics between breast cancer patients with/without visceral metastasis

Character	Training set				Validation set	
	Distant metastasis		HR (95% CI)	*P*-value	Distant metastasis	
	no	yes			no	yes
Age (mean ± SD, years)	47.1 (9.75)	46.3 (10.0)	0.984 (0.949–1.02)	0.395	48.9 (9.46)	48.3 (14.8)
Family history of BC						
Yes	4 (4.60%)	1 (3.33%)	0.985 (0.235–4.13)	0.983	2 (5.41%)	1 (8.33%)
No	83 (95.40%)	29 (96.67%)			35 (94.59%)	11 (91.67%)
Breast-feeding histories						
Yes	75 (86.21%)	26 (86.67%)	0.505 (0.216–1.18)	0.113	33 (89.19%)	8 (66.67%)
No	12 (13.79%)	4 (13.33%)			4 (10.81%)	4 (33.33%)
Abortion						
Yes	52 (59.77%)	18 (60.00%)	0.623 (0.303–1.28)	0.200	24 (64.86%)	3 (25.00%)
No	35 (40.23%)	12 (40.00%)			13 (35.14%)	9 (75.00%)
Reproductive history						
Yes	84 (96.55%)	28 (93.33%)	0.463 (0.162–1.33)	0.152	34 (91.89%)	9 (75.00%)
No	3 (3.45%)	2 (6.67%)			3 (8.11%)	3 (25.00%)
Menstrual status						
Menstruate	56 (64.37%)	20 (66.67%)	0.845 (0.396–1.81)	0.665	22 (59.46%)	6 (50.00%)
Menopause	31 (35.63%)	10 (33.33%)			15 (40.54%)	6 (50.00%)
Age of menarche	14.4 (1.69)	14.2 (1.69)	0.802 (0.635–1.01)	0.063	14.9 (1.79)	14.3 (2.38)
Lymph node metastasis						
Have	26 (29.89%)	19 (63.33%)	4.9 (2.29–10.5)	< 0.001	4 (10.81%)	7 (58.33%)
None	61 (70.11%)	11 (36.67%)			33 (89.19%)	5 (41.67%)
molecular subtyping						
1	9 (10.34%)	3 (10.00%)	1.08 (0.706–1.66)	0.718	4 (10.81%)	0 (0.00%)
2	61 (70.11%)	14 (46.67%)			24 (64.86%)	7 (58.33%)
3	4 (4.60%)	6 (20.00%)			2 (5.41%)	2 (16.67%)
4	13 (14.94%)	7 (23.33%)			7 (18.92%)	3 (25.00%)
ER						
Positive	70 (80.46%)	14 (46.67%)	0.456 (0.221–0.939)	0.033	28 (75.68%)	7 (58.33%)
Negative	17 (19.54%)	16 (53.33%)			9 (24.32%)	5 (41.67%)
PR						
Positive	69 (79.31%)	13 (43.33%)	0.482 (0.234–0.993)	0.048	26 (70.27%)	7 (58.33%)
Negative	18 (20.69%)	17 (56.67%)			11 (29.73%)	5 (41.67%)
HER2 status						
Positive	57	16	1.6 (1.02–2.5)	0.041	27	7
Negative	30	14			10	5
Ki-67						
Positive	77 (88.51%)	26 (86.67%)	0.996 (0.981–1.01)	0.603	32 (86.49%)	11 (91.67%)
Negative	10 (11.49%)	4 (13.33%)			5 (13.51%)	1 (8.33%)
TPSA						
Positive	8 (9.20%)	4 (13.33%)	0.596 (0.142–2.5)	0.479	5 (13.51%)	2 (16.67%)
Negative	79 (90.80%)	26 (86.67%)			32 (86.49%)	10 (83.33%)
CA153						
Positive	0 (0.00%)	5 (16.67%)	5.49 (2.09–14.4)	< 0.001	1 (2.70%)	3 (25.00%)
Negative	87 (100.00%)	25 (83.33%)			36 (97.30%)	9 (75.00%)
CEA						
Positive	1 (1.15%)	4 (13.33%)	5.52 (2.1–14.5)	0.004	0 (0.00%)	3 (25.00%)
Negative	86 (98.85%)	26 (86.67%)			37 (100.00%)	9 (75.00%)
CA125						
Positive	7 (8.05%)	4 (13.33%)	1.77 (0.616–5.07)	0.290	1 (2.70%)	2 (16.67%)
Negative	80 (91.95%)	26 (86.67%)			36 (97.30%)	10 (83.33%)
Operation						
No surgery	0 (0.00%)	3 (10.00%)	1.02 (0.503–2.05)	0.965	0 (0.00%)	2 (16.67%)
Conserving	40 (45.98%)	6 (20.00%)			15 (40.54%)	3 (25.00%)
Radical	47 (54.02%)	21 (70.00%)			22 (59.46%)	7 (58.33%)
Endocrinotherapy						
Yes	11 (12.64%)	0 (0.00%)	1.31e-08 (0-Inf)	0.997	1 (2.70%)	0 (0.00%)
No	76 (87.36%)	30 (100.00%)			36 (97.30%)	12 (100.00%)
Radiotherapy						
Yes	14 (16.09%)	8 (26.67%)	2.42 (1.08–5.45)	0.033	3 (8.11%)	3 (25.00%)
No	73 (83.91%)	22 (73.33%)			34 (91.89%)	9 (75.00%)
Chemotherapy						
Yes	78 (89.66%)	30 (100.00%)	3.02 (0.411–22.2)	0.277	35 (94.59%)	11 (91.67%)
No	9 (10.34%)	0 (0.00%)			2 (5.41%)	1 (8.33%)
RadScore (mean ± SD)	-2.77 (0.56)	-1.66 (0.44)	20 (8.27–48.2)	< 0.001	-2.60 (0.74)	-2.23 (0.60)

*Abbreviations: ER* Expression of the oestrogen receptor, *PR* Progesterone receptor, *HER2* Human epidermal growth factor receptor 2, *TPSA* Total prostate-specific antigen, *CA125* Carbohydrate antigen 125, *CEA* Carcinoembryonic antigen, *CA153* Carbohydrate antigen 125

**Table 4:** Clinicopathologic characteristics between breast cancer patients with/without brain metastasis

Character	All			
	Distant metastasis		HR (95% CI)	*P*-value
	no	yes		
Age (mean ± SD, years)	47.6 (9.66)	42.5 (6.22)	0.94 (0.874–1.01)	0.099
Family history of BC				
Yes	6 (4.84%)	0 (0.00%)	3.84e-08 (0-Inf)	0.998
No	118 (95.16%)	10 (100.00%)		
Breast-feeding histories				
Yes	108 (87.10%)	7 (70.00%)	0.375 (0.097–1.45)	0.156
No	16 (12.90%)	3 (30.00%)		
Marital status				
Married	122 (98.39%)	9 (90.00%)	0.214 (0.0271–1.69)	0.143
Never married	2 (1.61%)	1 (10.00%)		
Abortion				
Yes	76 (61.29%)	4 (40.00%)	0.44 (0.124–1.56)	0.203
No	48 (38.71%)	6 (60.00%)		
Reproductive history				
Yes	118 (95.16%)	9 (90.00%)	0.515 (0.0652–4.06)	0.528
No	6 (4.84%)	1 (10.00%)		
Menstrual status				
Menstruate	78 (62.90%)	8 (80.00%)	0.434 (0.0922–2.05)	0.292
Menopause	46 (37.10%)	2 (20.00%)		
Age of menarche	14.5 (1.73)	13.6 (1.51)	0.71 (0.465–1.08)	0.113
Lymph node metastasis				
Have	30 (24.19%)	5 (50.00%)	2.99 (0.866–10.3)	0.083
None	94 (75.81%)	5 (50.00%)		
molecular subtyping				
1	13 (10.48%)	0 (0.00%)	1.59 (0.856–2.97)	0.141
2	85 (68.55%)	6 (60.00%)		
3	6 (4.84%)	1 (10.00%)		
4	20 (16.13%)	3 (30.00%)		
ER				
Positive	98 (79.03%)	4 (40.00%)	0.197 (0.0557–0.7)	0.012
Negative	26 (20.97%)	6 (60.00%)		
PR				
Positive	95 (76.61%)	5 (50.00%)	0.33 (0.0956–1.14)	0.080
Negative	29 (23.39%)	5 (50.00%)		
HER2 status				
Positive	84 (67.74%)	6 (60.00%)	1.27 (0.559–2.9)	0.565
Negative	40 (32.26%)	4 (40.00%)		
Ki-67				
Positive	109 (87.90%)	10 (100.00%)	1.01 (0.983–1.03)	0.523
Negative	15 (12.10%)	0 (0.00%)		
TPSA				
Positive	13 (10.48%)	2 (20.00%)	2.12 (0.451–10)	0.341
Negative	111 (89.52%)	8 (80.00%)		
CA153				
Positive	1 (0.81%)	4 (40.00%)	35.7 (9.63–132)	< 0.001
Negative	123 (99.19%)	6 (60.00%)		
CEA				
Positive	1 (0.81%)	2 (20.00%)	18.2 (3.81–86.9)	< 0.001
Negative	123 (99.19%)	8 (80.00%)		
CA125				
Positive	8 (6.45%)	3 (30.00%)	5.5 (1.42–21.3)	0.013
Negative	116 (93.55%)	7 (70.00%)		
Operation				
No surgery	0 (0.00%)	2 (20.00%)	0.573 (0.177–1.86)	0.353
Conserving	55 (44.35%)	2 (20.00%)		
Radical	69 (55.65%)	6 (60.00%)		
Endocrinotherapy				
Yes	12 (9.68%)	0 (0.00%)	1.3e-08 (0-Inf)	0.998
No	112 (90.32%)	10 (100.00%)		
Radiotherapy				
Yes	17 (13.71%)	3 (30.00%)	2.66 (0.687–10.3)	0.157
No	107 (86.29%)	7 (70.00%)		
Chemotherapy				
Yes	113 (91.13%)	10 (100.00%)	75,600,000 (0-Inf)	0.998
No	11 (8.87%)	0 (0.00%)		
RadScore (mean ± SD)	-2.82 (0.53)	-1.72 (0.11)	0.356	< 0.001

*Abbreviations: ER* Expression of the oestrogen receptor, *PR* Progesterone receptor, *HER2* Human epidermal growth factor receptor 2, *TPSA* Total prostate-specific antigen, *CA125* Carbohydrate antigen 125, *CEA* Carcinoembryonic antigen, *CA153* Carbohydrate antigen 125

### Development and testing of the radiomics model

Four different feature sets were selected from the T2WI, DCE-MRI, US, and the combination of the imaging models. Through feature selection, 6, 4 and 1 features were selected from the T2WI, DCE-MRI, and US images, respectively. A total of 8 features were selected from the feature sets, including 6 features from T2WI and 2 features from DCE-MRI. These 8 features were used to build the optimal radiomics model based on the imaging models (Supplemental Materials and Methods).

The T2WI model yielded AUCs of 0.838 (95% CI: 0.753–0.923), 0.917 (95% CI: 0.859–0.975), 0.925 (95% CI: 0.869–0.981) in the training set and 0.792 (95% CI: 0.638–0.946), 0.794 (95% CI: 0.667–0.922), and 0.874 (95% CI: 0.777–0.972) in the validation set for 1-, 3-, and 5-year risk, respectively. The AUCs of the DCE-MRI model were 0.888 (95% CI: 0.816–0.960), 0.916 (95% CI: 0.870–0.961), 0.920 (95% CI: 0.876–0.964) in the training set and 0.729 (95% CI: 0.450–0.100), 0.800 (95% CI: 0.641–0.959), and 0.765 (95% CI: 0.619–0.911) in the validation set. The AUCs of the US model were 0.763 (95% CI: 0.655–0.871), 0.749 (95% CI: 0.662–0.835), 0.757 (95% CI: 0.673–0.842) in the training set and 0.567 (95% CI: 0.162–0.972), 0.538 (95% CI: 0.330–0.747), and 0.512 (95% CI: 0.328–0.696) in the validation set for 1-, 3-, and 5-year risk, respectively. The DeLong test showed that there was no significant difference between the AUCs of the training set and validation set in the four radiomics signature models (all *P* > 0.05). The comparative analysis among different models was shown in Supplementary Figure S2. There was no statistical significance between the combined model and the DCE-MRI model (all *P* > 0.05), although the AUC of the combined model was higher than that of the DCE-MRI model.

**Fig. 2:**
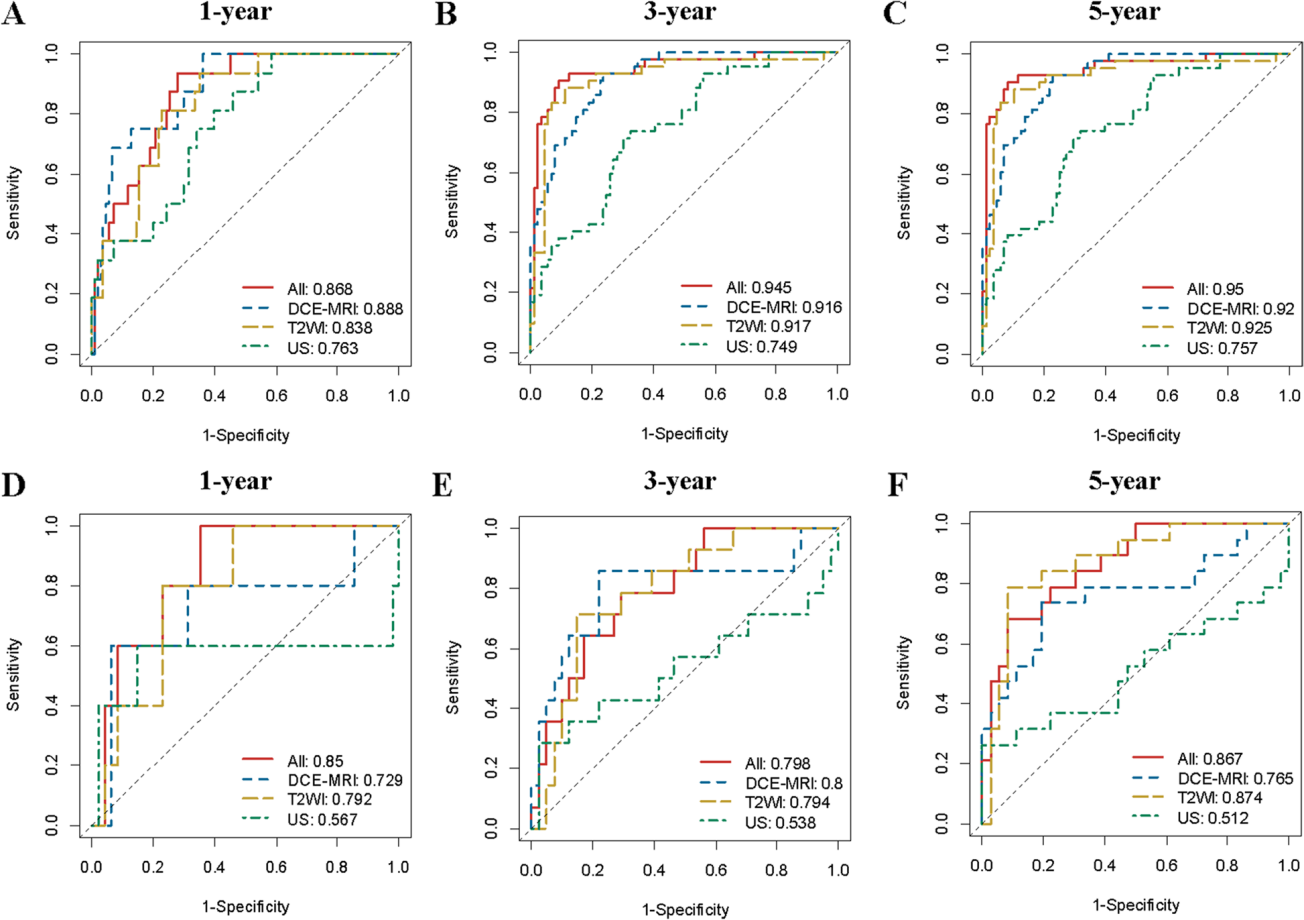
AUC of each radiomics model for 1-, 3-, and 5-year risk on training set (**A**-**C**) and validation set (**D**-**F**)

When T2WI, DCE-MRI, and US images were combined, the radiomics signature model with 8 features exhibited the highest AUC and obtained the best diagnostic accuracy. The AUCs were 0.868 (95% CI: 0.795–0.942), 0.945 (95% CI: 0.900–0.989), and 0.950 (95% CI: 0.907–0.993) in the training set and 0.850 (95% CI: 0.720–0.980), 0.798 (95% CI: 0.673–0.922), and 0.867 (95% CI: 0.772–0.962) in the validation set. The sensitivity, specificity, and AUC of each radiomics model are shown in Fig. 2 and Supplementary Table S3.

### Differences in the prediction performance between the clinicopathological-feature model and the clinicomics-based model for distant metastasis

**Fig. 3:**
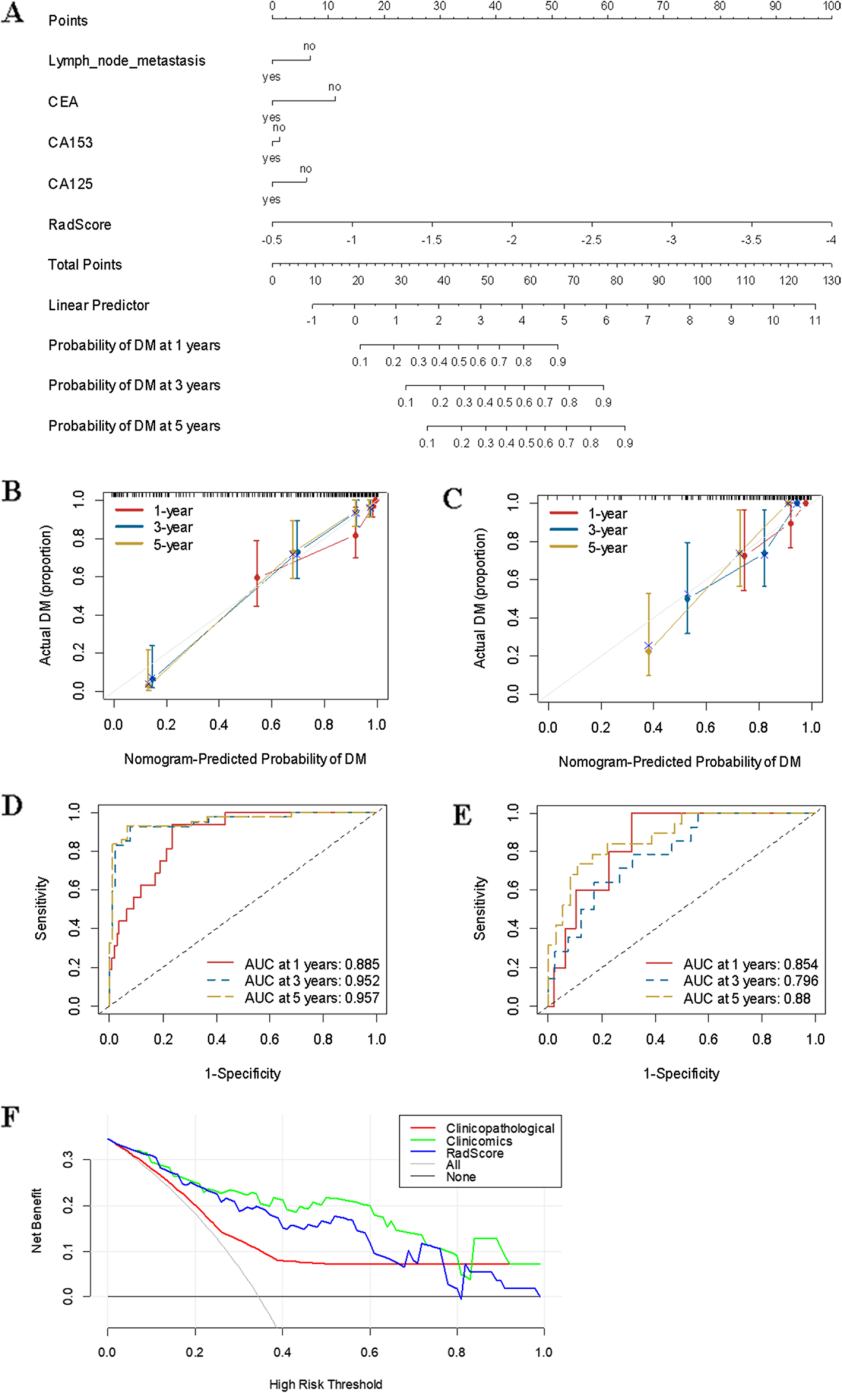
Construction of the clinicomics-based prediction model for non-distant metastasis (DM). **A** A nomogram was developed in the training data set with clinicopathological characteristics and RadScore. Calibration curves and ROC of the nomogram for the training set (**B** and **D**) and validation set (**C** and **E**). **F** Decision curve analysis derived from the validation cohort

As shown in Fig. 3, the clinicomics-based model provided a better performance in the training set (clinical: C-index = 0.725; clinicomics-based model: C-index = 0.882) and the validation set (clinical: C-index = 0.659; clinicomics-based model: C-index = 0.812). The areas under the curve (AUCs) at different follow-up times (1, 2, and 3 years) also confirmed that the clinicomics-based model had good prognostic accuracy in the training and validation sets. The calibration curves for the clinicomics-based model at 1 year, 2 years, and 3 years showed good agreement between the actual and predicted risk in the training and validation sets (Fig. 3). The clinicomics-based model showed a relatively better performance than the clinical model (IDI = 0.302, 95% CI: 0.174–0.431, *P* < 0.001).

The decision curve analysis revealed that the clinicomics-based nomogram had relatively good clinical performance compared with other models. These results suggested that the radiomic signature provided additional value for personalized DM prediction (Fig. 3F).

### Construction and validation of the predictive bone metastasis nomogram

**Fig. 4:**
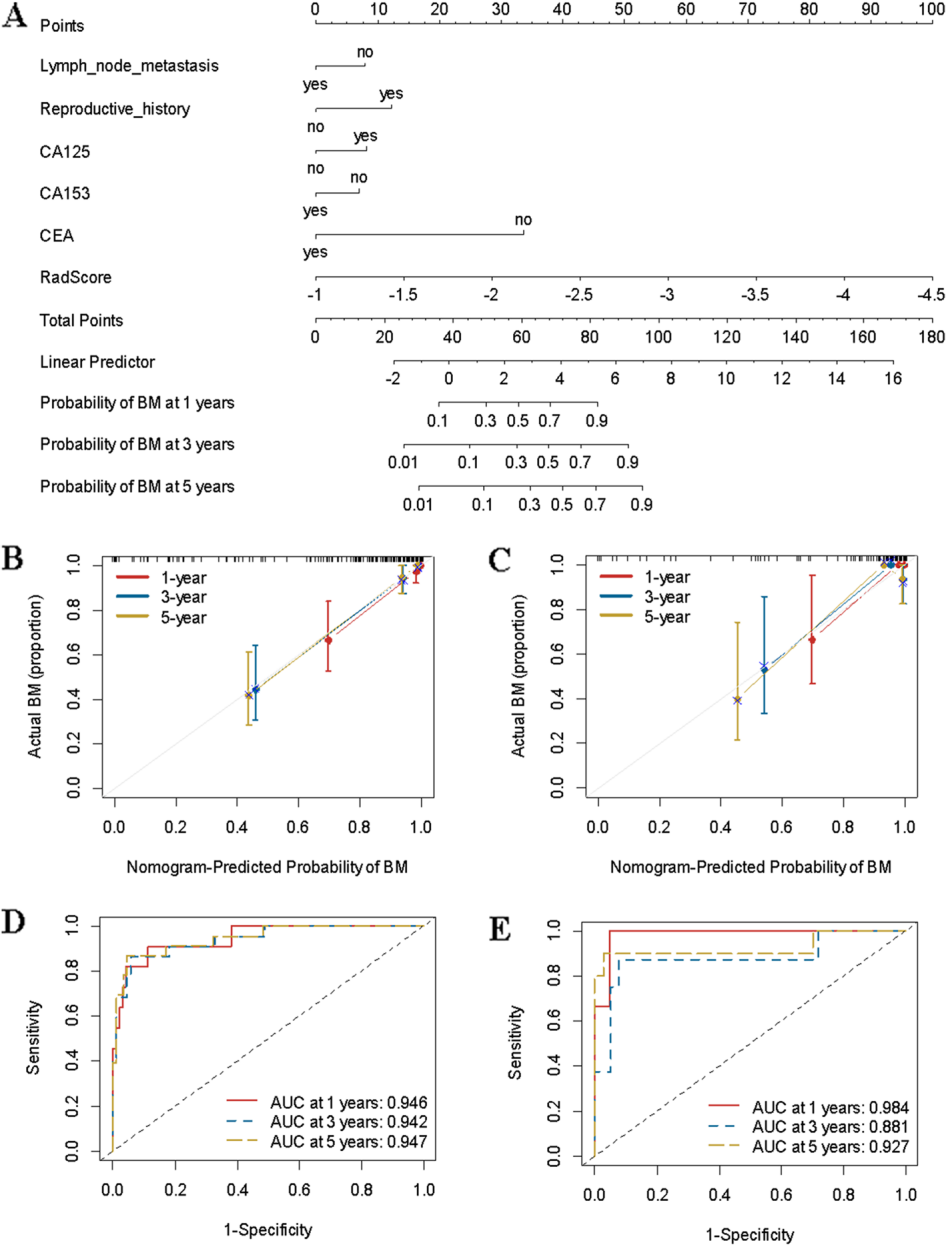
Construction of the clinicomics-based prediction model for bone metastasis (BM). **A** A nomogram was developed in the training data set with clinicopathological characteristics and RadScore. Calibration curves and ROC of the nomogram for the training set (**B** and **D**) and validation set (**C** and **E**)

A predictive bone metastasis nomogram was constructed, which included reproductive history, lymph node metastasis, CA153, CEA, CA125, and radiomics data (Fig. 4). The model showed good performance in both the primary (C index, 0.931; 95% CI: 0.868, 0.975) and validation cohorts (C index, 0.956; 95% CI: 0.926, 0.986). The ROC and calibration curves are shown in Fig. 4.

### Construction and validation of the predictive visceral metastasis nomogram

**Fig. 5:**
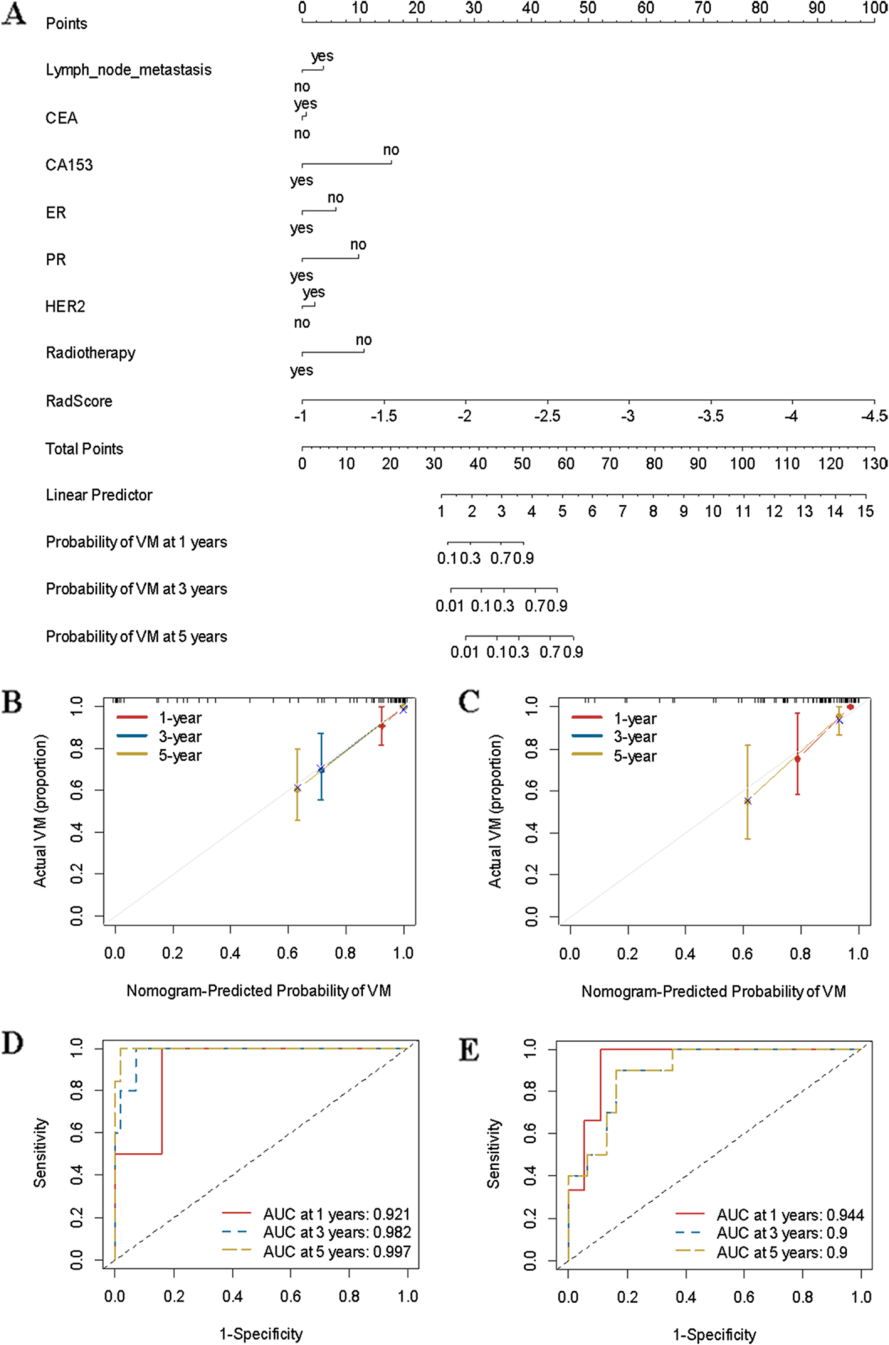
Construction of the clinicomics-based prediction model for visceral metastasis (VM). **A** A nomogram was developed in the training data set with clinicopathological characteristics and RadScore. Calibration curves and ROC of the nomogram for the training set (**B** and **D**) and validation set (**C** and **E**)

A predictive visceral metastasis nomogram was constructed, which included lymph node metastasis, CA153, CA153, ER, PR, HER2 and radiomics data (Fig. 5). The model showed good performance in both the primary (C index, 0.895; 95% CI: 0.850–0.941) and validation cohorts (C index, 0.946; 95% CI: 0.918–0.975). The ROC and calibration curves are shown in Fig. 5.

## Discussion

Distant metastasis in breast cancer can be divided into two types based on DM diagnosis: synchronous DM at initial and metachronous DM that arises later in the disease course. There is sufficient evidence that the significant difference between synchronous DM and metachronous DM can be found, including clinicopathologic characteristics, treatment responses and survival outcomes [12–14]. Compared with patients with synchronous DM, patients with metachronous DM usually show better survival outcomes [15]. Two main potential explanations were summarized: 1. BC patients with synchronous DM usually visit the hospital later than patients with metachronous DM. 2. Treatment response is usually worse in BC patients with synchronous DM due to faster BC progression [16]. Few studies of metachronous DM prediction in BC have been performed, and conducting such studies may guide individualized DM screening during the disease course.

This is the first study to investigate and validate DM risk prediction through cliniomics in BC patients. The concept of “omics” has been widely studied and applied, including genomics, proteomics, radiomics, and metabolomics [17, 18]. “Omics” are developed based on patterns of changes in complex processes. Thus, this concept and approach can also be applied to patients in the clinic using the multidimensional features (complete history, epidemiological distribution, physical examination, laboratory test, imaging evaluation and histological examination) that are routinely investigated in a clinical evaluation of a patient. Before the development of AI techniques, clinicomics remained a hypothesis due to the difficulty of imaging/video data dimension reduction and integration.

Breast US, with a proper balance of specificity and sensitivity, is widely accepted as the first choice for breast lesion evaluation [19–21]. Compared with other imaging evaluations, MRI has the highest sensitivity for invasive lesion detection, and such sensitivity is not impaired by fibroglandular tissue, fibrous scarring, radiotherapy, breast implants, or other breast reconstruction [19, 22, 23]. A recent study combined clinical features and MRI features and suggested that this method performs well for brain metastasis prediction before radiosurgery [24]. MRI features were suggested to be of significance for DM prediction in locally advanced rectal cancer [25]. Our previous study verified the significance of MRI for DM prediction in BC [26]. In the latest study, the combination of MRI and US showed satisfactory prediction ability for residual tumour size in early breast cancer [27]. A study evaluating the accuracy of various imaging methods in BC concluded that MRI had advantages for evaluating suspicious breast lumps but had low specificity. US was able to compensate for MRI’s low specificity in image formation [28]. Therefore, we combined the features from MRI and US to develop a clinicomics approach to DM prediction in BC patients.

The most important finding of the present study was that we created and validated DM prediction in BC through clinicomics. This approach can potentially be used in various clinical fields. The features extracted from the high-dimensional images can provide additional information. Factors including complete history, epidemiological distribution, physical examination, and laboratory tests can reflect each patient’s reaction to the specific tumour. Thus, a comprehensive judgement can be reached using AI-guided clinicomics analyses, and such analyses have potential applications.

Three prediction models were created to respectively predict DM risk, bone metastatic risk and visceral metastasis risk. Each model showed a good ability to predict DM in BC, which could be used to stratify BC patients into different groups according to their risk for DM. Among the created models, the DM prediction model can be used for survival evaluation and general DM screening. A bone metastasis prediction model is of significance for the prevention and treatment of bone metastasis and potentially reduces adverse skeletal-related events. A visceral metastasis prediction model can guide DM screening of viscera through imaging examination and reduce unnecessary radiation exposure.

Our study has some limitations. First, the external validation with large population and various human ethnicities will be needed. Second, further studies will be needed to analyse the effect of incorporating other imaging data into the predictive nomogram, such as mammography. Finally, several serological biomarkers indicating metastasis potential of tumors such as EZH2 and PDGF were not analyzed in the study [29].

## Conclusion

We validated the importance of clinicomics for predicting the risk for DM and organ-specific DM in BC. Three AI-guided clinicomics prediction models in BC were created: (1) the DM prediction model, (2) the bone metastasis prediction model, and (3) the visceral metastasis prediction model. These models can potentially guide metachronous DM screening and the implementation of individualized therapy in BC. AI-guided clinicomics strategies possess the potential for wide application in the clinic.

## Supplementary Information

**Additional file 1: Supplemental Materials and Methods.** **Supplementary Table S1.** Texture features used in this study. **Supplementary Table S2.** Clinicopathologic characteristics between breast cancer patients with /without distant metastasis in different age groups (Training set). **Supplementary Table S3.** Performance comparison among radiomics model in the training and validation set of different image types. **Supplementary Figure S1**. Construction of the clinicopathological model for predicting distant metastasis (DM). (A) A nomogram was developed in the training data set with clinicopathological characteristics. Calibration curves and ROC of the nomogram for the training set (B and D) and validation set (C and E). **Supplementary Figure S2.** The difference analysis among different radiomics signature models with DeLong test in training set (A-C) and validation set (D-F).

## Data Availability

The related data and materials are available for sharing upon request to Wenjuan Ma and Chao Zhang.

## Abbreviations

DM: Distant Metastasis
BC: Breast Cancer
MRI: Magnetic Resonance Imaging
ER: Estrogen Receptor
PR: Progesterone Receptor
T2WI: T2-Weighted Imaging
LASSO: Least Absolute Shrink Age and Selection Operator
ROC: Receiver operating characteristic
AUC: Area Under the Curve
IDI: Integrated Discrimination Improvement
NRI: Net Reclassification Improvement
