# Supplementary material for: Clinicomics-guided distant metastasis prediction in breast cancer via artificial intelligence
Source: BMC Cancer. 2023 Mar 14;23:239. doi: 10.1186/s12885-023-10704-w (PMC10012565; doi:10.1186/s12885-023-10704-w)
Supplement: Supplementary file 1 — Additional file 1: Supplemental Materials and Methods. Supplementary Table S1. Texture features used in this study. Supplementary Table S2. Clinicopathologic characteristics between breast cancer patients with /without distant metastasis in different age groups (Training set). Supplementary Table S3. Performance comparison among radiomics model in the training and validation set of different image types. Supplementary Figure S1. Construction of the clinicopathological model for predicting distant metastasis (DM). (A) A nomogram was developed in the training data set with clinicopathological characteristics. Calibration curves and ROC of the nomogram for the training set (B and D) and validation set (C and E). Supplementary Figure S2. The difference analysis among different radiomics signature models with DeLong test in training set (A-C) and validation set (D-F). [file 12885_2023_10704_MOESM1_ESM.docx]

***Supplemental Materials and Methods***

1. ***MRI technique***

Magnetic resonance images were acquired at the Tianjin Medical University Cancer Institute & Hospital using scanners manufactured by two different companies. MRI was performed with a 1.5-T system equipped with a dedicated four-channel phased-array bilateral breast coil (Signa Infinity Excite II, GE Healthcare) before 2013, while a 3.0-T MRI system equipped with a dedicated eight-channel phased-array breast coil (Discovery MR750, GE Medical Systems) was used after 2013. Some examinations were performed with a 3.0-T scanner using a dedicated 8-channel (4-channel for 1.5T scanner) phased-array breast coil. MRI protocols included axial T1-weighted imaging, fat-saturated fast spin-echo (FSE) sequences for T2-weighted imaging (T2WI) and unilateral sagittal fat-saturated FSE T2-weighted imaging of the affected breast before contrast administration. Diffusion-weighted imaging (DWI) was performed using a multi-section spin-echo single-shot echo-planar sequence bilaterally in the axial plane and in the sagittal plane of the affected breast. Images and sagittal data were obtained by sagittal DCE-MRI using the volume imaging for breast assessment (VIBRANT) bilateral breast imaging technique. Before the injection of the contrast agent, serial mask images were obtained. A contrast agent (Gd-DTPA, 0.2 mL/kg body weight, flow rate 2.0 mL/s) was manually injected using an automatic MR-compatible power injector and then flushed with the same total dose of saline solution. Dynamic MRI was immediately performed after the injection. Image acquisition was repeated five times (eight times for the 1.5T scanner), and each phase took 90-100 seconds (58-62 seconds for the 1.5T scanner). In all patients, final axial 3D fast spoiled gradient-recalled echo images were obtained after the dynamic study.

1. ***Image feature extraction***

To normalize the different image specifications from various US scanners, image resampling and gray-level normalization were performed before quantitative feature extraction. All image data were resampled at a 1×1-mm (US images) or 1×1×1-mm (DCE-MRI and T2 US images) voxel space size. The quantitative features were extracted from ROIs using an in-house software developed with MATLAB 2018B (MathWorksInc.).

A total of 855 radiomic features of US images and 858 radiomics features of DCE-MRI/T2 images were drawn from each segmented lesion and can grouped as follows: (1) Morphologic features: four metrics, including area, largest diameter, length to width ratio and roundness, were calculated for the morphological description of the US images. Seven metrics, including eccentricity, solidity, volume, surface area, compactness, surface to volume ratio and spherictiy, were calculated for the morphological description of the MRI images. (2) Gray-scale histograms features: three features were computed for each lesion according to the definitions of the gray-scale histogram: variance, skewness, and kurtosis. Their definition can be found in literatures Rodenacker K (2003). (3) Texture features: in total, 40 texture features were extracted from the tumor regions of US images after wavelet transform. Table S1 presents the list of texture features used in this study. Detailed description and methodology employed to extract the texture features is available in M Vallières (2015). (4) Wavelet features: wavelet transform effectively decouples textural information by decomposing the original image. In this study a discrete, one-level and undecimated two-dimensional wavelet transform was applied to each US image, which decomposes the original image into 4 decompositions (LL, HL, LH and HH). Undecimated three-dimensional wavelet transform was applied to each DCE-MRI/T2 image, which decomposes the original image into 8 decompositions (LLH, LHL, LHH, HLL, HLH, HHL, HHH, LLL). For each decomposition we computed gray-scale histograms and the textural features as described in Table S1.

Supplementary Table S1. Texture features used in this study.

| Texture type | Reference(s) | Texture name |
| --- | --- | --- |
| GLCM^a^ | (Haralick et al 1973) | Energy |
|  |  | Contrast |
|  |  | Correlation |
|  |  | Homogeneity |
|  |  | Variance |
|  |  | Sum Average |
|  |  | Entropy |
|  |  | Dissimilarity |
|  |  | Auto Correlation |
| GLRLM^b^ | (Galloway 1975) | Short Run Emphasis (SRE) |
|  |  | Long Run Emphasis (LRE) |
|  |  | Gray-Level Non-uniformity (GLN) |
|  |  | Run-Length Non-uniformity (RLN) |
|  |  | Run Percentage (RP) |
|  | (Chu et al 1990) | Low Gray-Level Run Emphasis (LGRE) |
|  |  | High Gray-Level Run Emphasis (HGRE) |
|  | (Dasarathy and Holder 1991) | Short Run Low Gray-Level Emphasis (SRLGE) |
|  |  | Short Run High Gray-Level Emphasis (SRHGE) |
|  |  | Long Run Low Gray-Level Emphasis (LRLGE) |
|  |  | Long Run High Gray-Level Emphasis (LRHGE) |
|  | (Thibault et al 2009) | Gray-Level Variance (GLV) |
|  |  | Run-Length Variance (RLV) |
| GLSZM^c^ | (Galloway 1975, Thibault et al 2009) | Small Zone Emphasis (SZE) |
|  |  | Large Zone Emphasis (LZE) |
|  |  | Gray-Level Non-uniformity (GLN) |
|  |  | Zone-Size Non-uniformity (ZSN) |
|  |  | Zone Percentage (ZP) |
|  | (Chu et al 1990, Thibault et al 2009) | Low Gray-Level Zone Emphasis (LGZE) |
|  |  | High Gray-Level Zone Emphasis (HGZE) |
|  | (Dasarathy and Holder 1991, | Small Zone Low Gray-Level Emphasis (SZLGE) |
|  | Thibault et al 2009) | Small Zone High Gray-Level Emphasis (SZHGE) |
|  |  | Large Zone Low Gray-Level Emphasis (LZLGE) |
|  |  | Large Zone High Gray-Level Emphasis (LZHGE) |
|  | (Thibault et al 2009) | Gray-Level Variance (GLV) |
|  |  | Zone-Size Variance (ZSV) |
| NGTDM^d^ | (Amadasun and King 1989) | Coarseness |
|  |  | Contrast |
|  |  | Busyness |
|  |  | Complexity |
|  |  | Strength |

^a^ GLCM: Gray-level co-occurence matrix.

^b^ GLRLM: Gray-level run-length matrix.

^c^ GLSZM: Gray-level size zone matrix.

^d^ NGTDM: Neighborhood gray-tone difference matrix.

1. ***Radiomics score***

*All*

Rad-score was calculated by summing the selected features weighted by their coefficients. The final formula of rad-score is:

*RadScore=1-0.230638905***T2WI_text_Ng_161_glszm_SZE-1.512926880*T2WI_wave_glszm_8_LL_SZE+0.905086831*T2WI_wave_glrlm_8_HH_GLN+0.031834732*T2WI_wave_glo_16_HL_Kurtosis-0.002699764*T2WI_wave_ngtdm_32_HH_Complexity-0.477871618*T2WI_wave_glszm_64_HH_ZSN-1.263865160*DCE_wave_glszm_8_HH_SZLGE-9.178492431*DCE_wave_ngtdm_64_LL_Coarseness*

*DCE*

Rad-score calculation formula:

*Radscore=1+3.472684e-07*DCE_volume-2.175612e+03*DCE_text_Ng_161_glszm_ZSV-1.260316e+01*DCE_wave_glrlm_8_LL_GLV+5.823855e-01*DCE_wave_glrlm_8_HL_SRE-1.024764e+00*DCE_wave_glszm_8_HH_SZLGE-4.196462e-01*DCE_wave_glrlm_16_HL_LRLGE*

And we compared the Rad-scores from two class on training group and test group respectively. The cutoff value was: 0.491.

*T2WI*

Rad-score calculation formula:

*Radscore=1-1.170468113*T2WI_wave_glszm_8_LL_SZE-0.012678326*T2WI_wave_glszm_16_LL_SZE-0.002910373*T2WI_wave_ngtdm_32_HH_Complexity-1.625893166*T2WI_wave_glszm_64_HH_SZE*

*US*

Rad-score calculation formula:

*Radscore=1+0.002851138*US_wave_glo_64_HH_Kurtosis*

Supplementary Table S2. Clinicopathologic characteristics between breast cancer patients with /without distant metastasis in different age groups (Training set).

| Character | **Age <50 years** | | | | **Age ≥ 50 years** | | | |
| --- | --- | --- | --- | --- | --- | --- | --- | --- |
|  | Distant metastasis | | HR  (95% CI) | *P*-value | Distant metastasis | | HR  (95% CI) | *P*-value |
|  | No | Yes |  |  | No | Yes |  |  |
| Family history of BC |  |  |  |  |  |  |  |  |
| Yes | 1 (1.8) | 1 (4.8) | 2.242  (0.3-16.76) | 0.432 | 2 (6.5) | 1 (4.5) | 0.697  (0.094-5.187) | 0.725 |
| No | 56 (98.2) | 20 (95.2) |  |  | 29 (93.5) | 21 (95.5) |  |  |
| Breast-feeding histories |  |  |  |  |  |  |  |  |
| Yes | 50 (87.7) | 14 (66.7) | 0.389  (0.157-0.966) | 0.042 | 27 (87.1) | 21 (95.5) | 2.748  (0.369-20.448) | 0.323 |
| No | 7 (12.3) | 7 (33.3) |  |  | 4 (12.9) | 1 (4.5) |  |  |
| Abortion |  |  |  |  |  |  |  |  |
| Yes | 35 (61.4) | 11 (52.4) | 0.751  (0.319-1.769) | 0.513 | 20 (64.5) | 10 (45.5) | 0.545  (0.235-1.263) | 0.157 |
| No | 22 (38.6) | 10 (47.6) |  |  | 11 (35.5) | 12 (54.5) |  |  |
| Reproductive history |  |  |  |  |  |  |  |  |
| Yes | 54 (94.7) | 16 (76.2) | 0.326  (0.119-0.892) | 0.029 | 31 (100) | 22 (100) | - | - |
| No | 3 (5.3) | 5 (23.8) |  |  | 0 | 0 |  |  |
| Menstrual status |  |  |  |  |  |  |  |  |
| Menstruate | 54 (94.7) | 19 (90.5) | 1.715  (0.399-7.371) | 0.468 | 3 (9.7) | 4 (18.2) | 0.674  (0.228-1.995) | 0.476 |
| Menopause | 3 (5.3) | 2 (9.5) |  |  | 28 (90.3) | 18 (81.8) |  |  |
| Lymph node metastasis |  |  |  |  |  |  |  |  |
| Have | 13 (22.8) | 12 (57.1) | 3.429  (1.441-8.162) | 0.005 | 5 (16.1) | 14 (63.6) | 4.94  (2.054-11.881) | <0.001 |
| None | 44 (77.2) | 9 (42.9) |  |  | 26 (83.9) | 8 (36.4) |  |  |
| Molecular subtyping |  |  |  |  |  |  |  |  |
| Luminal A | 4 (7) | 3 (14.3) | 1.181  (0.74-1.886) | 0.485 | 5 (16.1) | 2 (9.1) | 1.042  (0.678-1.6) | 0.852 |
| Luminal B | 42 (73.7) | 10 (47.6) |  |  | 17 (54.8) | 13 (59.1) |  |  |
| HER2-enriched | 2 (3.5) | 3 (14.3) |  |  | 3 (9.7) | 3 (13.6) |  |  |
| Triple-negative | 9 (15.8) | 5 (23.8) |  |  | 6 (19.4) | 4 (18.2) |  |  |
| ER |  |  |  |  |  |  |  |  |
| Positive | 46 (80.7) | 13 (61.9) | 0.5  (0.207-1.208) | 0.124 | 22 (71) | 14 (63.6) | 0.89  (0.373-2.125) | 0.793 |
| Negative | 11 (19.3) | 8 (38.1) |  |  | 9 (29) | 8 (36.4) |  |  |
| PR |  |  |  |  |  |  |  |  |
| Positive | 45 (78.9) | 12 (57.1) | 0.455  (0.191-1.081) | 0.074 | 20 (64.5) | 13 (59.1) | 0.881  (0.376-2.063) | 0.771 |
| Negative | 12 (21.1) | 9 (42.9) |  |  | 11 (35.5) | 9 (40.9) |  |  |
| HER2 status |  |  |  |  |  |  |  |  |
| Positive | 19 (33.3) | 10 (47.6) | 1.672  (0.71-3.938) | 0.24 | 8 (25.8) | 7 (31.8) | 1.17  (0.477-2.872) | 0.732 |
| Negative | 38 (66.7) | 11 (52.4) |  |  | 23 (74.2) | 15 (68.2) |  |  |
| Ki-67 |  |  |  |  |  |  |  |  |
| Positive | 52 (91.2) | 17 (81) | 0.428  (0.144-1.273) | 0.127 | 26 (83.9) | 21 (95.5) | 3.006  (0.404-22.366) | 0.282 |
| Negative | 5 (8.8) | 4 (19) |  |  | 5 (16.1) | 1 (4.5) |  |  |
| TPSA |  |  |  |  |  |  |  |  |
| Positive | 6 (10.5) | 3 (14.3) | 1.36  (0.4-4.622) | 0.622 | 4 (12.9) | 3 (13.6) | 1.125  (0.333-3.806) | 0.849 |
| Negative | 51 (89.5) | 18 (85.7) |  |  | 27 (87.1) | 19 (86.4) |  |  |
| CA153 |  |  |  |  |  |  |  |  |
| Positive | 0 (0) | 4 (19) | 19.742  (5.594-69.675) | <0.001 | 1 (3.2) | 6 (27.3) | 3.835  (1.478-9.951) | 0.006 |
| Negative | 57 (100) | 17 (81) |  |  | 30 (96.8) | 16 (72.7) |  |  |
| CEA |  |  |  |  |  |  |  |  |
| Positive | 0 (0) | 2 (9.5) | 9.954  (2.212-44.791) | 0.003 | 0 (0) | 7 (31.8) | 7.718  (2.949-20.199) | <0.001 |
| Negative | 57 (100) | 19 (90.5) |  |  | 31 (100) | 15 (68.2) |  |  |
| CA125 |  |  |  |  |  |  |  |  |
| Positive | 2 (3.5) | 3 (14.3) | 3.292  (0.968-11.201) | 0.056 | 2 (6.5) | 5 (22.7) | 2.653  (0.974-7.228) | 0.056 |
| Negative | 55 (96.5) | 18 (85.7) |  |  | 29 (93.5) | 17 (77.3) |  |  |
| Operation |  |  |  |  |  |  |  |  |
| No surgery | 0 (0) | 3 (14.3) | 1.304  (0.572-2.975) | 0.528 | 0 (0) | 4 (18.2) | 0.532  (0.287-0.986) | 0.045 |
| Conserving | 30 (52.6) | 3 (14.3) |  |  | 9 (29) | 5 (22.7) |  |  |
| Radical | 27 (47.4) | 15 (71.4) |  |  | 22 (71) | 13 (59.1) |  |  |
| Endocrinotherapy |  |  |  |  |  |  |  |  |
| Yes | 8 (14) | 0 (0) | 0.041  (0-13.634) | 0.281 | 3 (9.7) | 0 (0) | 0.044  (0-56.603) | 0.393 |
| No | 49 (86) | 21 (100) |  |  | 28 (90.3) | 22 (100) |  |  |
| Radiotherapy |  |  |  |  |  |  |  |  |
| Yes | 10 (17.5) | 7 (33.3) | 1.952  (0.787-4.845) | 0.149 | 3 (9.7) | 3 (13.6) | 1.128  (0.333-3.815) | 0.847 |
| No | 47 (82.5) | 14 (66.7) |  |  | 28 (90.3) | 19 (86.4) |  |  |
| Chemotherapy |  |  |  |  |  |  |  |  |
| Yes | 52 (91.2) | 21 (100) | 22.585(0.016-31230.11) | 0.398 | 27 (87.1) | 21 (95.5) | 2.678  (0.36-19.924) | 0.336 |
| No | 5 (8.8) | 0 (0) |  |  | 4 (12.9) | 1 (4.5) |  |  |
| RadScore (mean±SD) | -2.81 (0.52) | -1.62 (0.48) | 14.644  (5.893-36.390) | <0.001 | -2.88 (0.44) | -1.71 (0.43) | 6.593  (3.253-13.361) | <0.001 |

Abbreviations: ER: Expression of the oestrogen receptor; PR: Progesterone receptor; HER2: Human epidermal growth factor receptor 2; TPSA: Total prostate-specific antigen; CA125: Carbohydrate antigen 125; CEA: Carcinoembryonic antigen; CA153: Carbohydrate antigen 125.

| Supplementary Table S3. Performance comparison among radiomics model in the training and validation set of different image types | | | | | | | |
| --- | --- | --- | --- | --- | --- | --- | --- |
|  |  | Training cohort | | | Validation cohort | | |
|  |  | AUC (95% CI) | SEN | SPN | AUC (95% CI) | SEN | SPN |
| 1 year- | DCE-MRI | 0.888 (0.816,0.960) | 1 | 0.640 | 0.729 (0.450, 0.100) | 0.6 | 0.938 |
|  | T2WI | 0.838 (0.753, 0.923) | 0.812 | 0.775 | 0.792 (0.638, 0.946) | 0.8 | 0.771 |
|  | US | 0.763 (0.655,0.871) | 0.813 | 0.604 | 0.567 (0.162, 0.972) | 0.6 | 0.854 |
|  | All | 0.868 (0.795, 0.942) | 0.938 | 0.721 | 0.850 (0.720, 0.980) | 1 | 0.646 |
| 3 year- | DCE-MRI | 0.916 (0.870, 0.961) | 0.929 | 0.764 | 0.800 (0.641, 0.959) | 0.857 | 0.780 |
|  | T2WI | 0.917 (0.859, 0.975) | 0.881 | 0.888 | 0.794 (0.667, 0.922) | 0.714 | 0.853 |
|  | US | 0.749 (0.662, 0.835) | 0.738 | 0.674 | 0.538 (0.330, 0.747) | 0.286 | 0.976 |
|  | All | 0.945 (0.900, 0.989) | 0.905 | 0.910 | 0.798 (0.673, 0.922) | 0.786 | 0.707 |
| 5 year- | DCE-MRI | 0.920 (0.876, 0.964) | 0.930 | 0.773 | 0.765 (0.619, 0.911) | 0.736 | 0.805 |
|  | T2WI | 0.925 (0.869, 0.981) | 0.884 | 0.898 | 0.874 (0.777, 0.972) | 0.789 | 0.916 |
|  | US | 0.757 (0.673, 0.842) | 0.744 | 0.682 | 0.512 (0.328, 0.696) | 0.263 | 1 |
|  | All | 0.950 (0.907, 0.993) | 0.907 | 0.920 | 0.867 (0.772, 0.962) | 0.684 | 0.917 |
| AUC area under the receiver operator characteristic curves, SEN sensitivity, SPEC specificity. | | | | | | | |

1. **Supplementary figures**

Supplementary Figure S1. Construction of the clinicopathological model for predicting distant metastasis (DM). (A) A nomogram was developed in the training data set with clinicopathological characteristics. Calibration curves and ROC of the nomogram for the training set (B and D) and validation set (C and E) .

Supplementary Figure S2. The difference analysis among different radiomics signature models with DeLong test in training set (A-C) and validation set (D-F).
